# Electrophysiological activity from over the cerebellum and cerebrum during eye blink conditioning in human subjects

**DOI:** 10.14814/phy2.15642

**Published:** 2023-03-27

**Authors:** Neil P. M. Todd, Sendhil Govender, Peter E. Keller, James G. Colebatch

**Affiliations:** ^1^ Department of Psychology University of Exeter Exeter UK; ^2^ School of Clinical Medicine, Randwick Campus UNSW Sydney New South Wales Australia; ^3^ Neuroscience Research Australia UNSW Sydney New South Wales Australia; ^4^ MARCS Institute for Brain, Behaviour and Development Western Sydney University Penrith New South Wales Australia; ^5^ Center for Music in the Brain, Department of Clinical Medicine Aarhus University Aarhus Denmark

**Keywords:** cerebellum, climbing fiber response, electrocerebellogram, eye blink conditioning

## Abstract

We report the results of an experiment in which electrophysiological activity was recorded from the human cerebellum and cerebrum in a sample of 14 healthy subjects before, during and after a classical eye blink conditioning procedure with an auditory tone as conditional stimulus and a maxillary nerve unconditional stimulus. The primary aim was to show changes in the cerebellum and cerebrum correlated with behavioral ocular responses. Electrodes recorded EMG and EOG at peri‐ocular sites, EEG from over the frontal eye‐fields and the electrocerebellogram (ECeG) from over the posterior fossa. Of the 14 subjects half strongly conditioned while the other half were resistant. We confirmed that conditionability was linked under our conditions to the personality dimension of extraversion‐introversion. Inhibition of cerebellar activity was shown prior to the conditioned response, as predicted by Albus (1971). However, pausing in high frequency ECeG and the appearance of a contingent negative variation (CNV) in both central leads occurred in all subjects. These led us to conclude that while conditioned cerebellar pausing may be necessary, it is not sufficient alone to produce overt behavioral conditioning, implying the existence of another central mechanism. The outcomes of this experiment indicate the potential value of the noninvasive electrophysiology of the cerebellum.

## Graphical abstract

We report the results of an experiment in which electrophysiological activity was recorded from the human cerebellum and cerebrum in a sample of 14 healthy subjects before, during and after a classical eye‐blink conditioning procedure with an auditory tone as conditional stimulus and a maxillary nerve unconditional stimulus. The primary aim was to show changes in the cerebellum and cerebrum correlated with behavioural ocular responses.

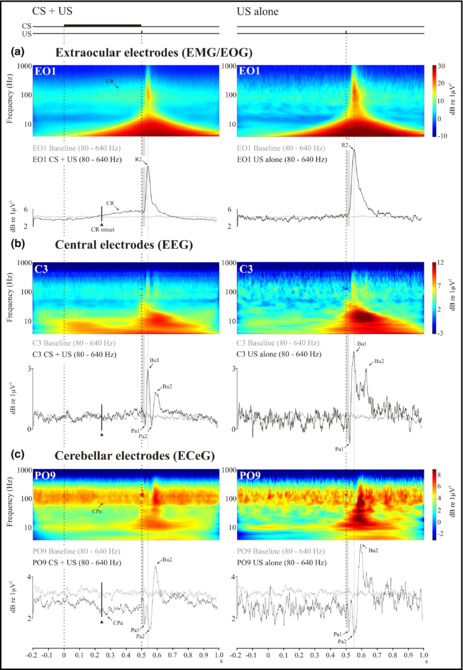

## 1 INTRODUCTION

The cerebellum is accepted to have a crucial role in classical conditioning. Lobule HVI is particularly implicated in eye blink conditioning (e.g., Villarreal and Steinmetz, 2005; Jirenhed and Hesslow, 2016). Learning is still possible after lesions of the cerebellar cortex, but is delayed and poor quality (Lavond and Steinmetz, 1989). In this simple form of associative learning the pairing of a conditional stimulus (CS), such as a tone, with an unconditional stimulus (US), such as an air puff, which produces an eye blink unconditioned response (UR), results in the acquisition of a conditioned response (CR) with an onset prior to the US.

Following the classical work of Eccles et al. (1967) in elucidating its detailed neurophysiology, several theorists were inspired to develop computational models of cerebellar learning (Marr, 1969; Albus, 1971). In Albus' original conception, the so‐called “inactivation response” of a Purkinje cell (PC), a pause in PC spontaneous activity of about 15–30 ms associated with a climbing fiber response (CFR), could be interpreted as the internal neural representation of the overt UR. He further suggested that mossy/parallel fiber activity produced by the CS could be considered its internal neural representation. The effect of learning, by changing PF‐PC synaptic weights with conjunctive CF/PF inputs, and the acquisition of a CR would, he hypothesized, be accompanied by a conditioned pause of Purkinje neurones, thus disinhibiting their target cerebellar nuclei and facilitating transmission through them.

Direct recordings from animal models have since provided evidence to support the Albus hypothesis by demonstrating Purkinje activity being transiently silenced in response to learning (e.g., Hesslow, 1994; Yeo and Hardiman, 1992; Jirenhed and Hesslow, 2016; Yeo and Hesslow, 1998). There is also strong evidence from the effects of lesions in humans that the cerebellum is required for the acquisition of classically conditioned eye blink responses (e.g., Lye et al., 1988; Topka et al., 1993; Gerwig et al., 2007). The association is such that the acquisition of eye blink conditioning is used as a test of human cerebellar function (Gerwig et al., 2007; Teo et al., 2009). However, it is also widely recognized that the original Marr‐Albus‐Ito conception is inadequate for several reasons (Sanger et al., 2020; Kawato et al., 2021), in part because of its conception of cerebellar learning in isolation from other cerebral structures also implicated in motor learning. These include the motor cortex (López‐Ramos and Delgado‐García, 2021), the hippocampus (Delgado‐García and Gruart, 2006), the prefrontal cortex (Wu et al., 2012) and basal ganglia (Gillies and Arbuthnott, 2000). Further, in the case of human eye blink conditioning it is known that individuals vary considerably in their conditionability, this variability in conditioning forming an important empirical basis for testing theories of the neural basis of individual differences (Evans and Wilson, 2016). Indeed, it has been established that the personality factor of extraversion‐introversion is predictive of conditionability for non‐nociceptive USs (Eysenck, 1965; Eysenck and Levey, 1972). There is also a critical debate on the role of contingency awareness in human conditioning (Weidemann et al., 2016). Thus, while the cerebellar learning mechanisms may be necessary, they may not be sufficient to explain human conditioning. More fundamentally, it remains to be shown that the unconditional and conditional changes in cerebellar activity accompanying classical conditioning can be demonstrated noninvasively in humans.

It had been widely thought that the cerebellum is particularly difficult to record from noninvasively (Andersen et al., 2020). The reasons postulated for the difficulty in recording cerebellar activity have included the fine folding of the cerebellum, potentially canceling potentials seen at a distance, or excess EMG noise from the musculature of the neck. Nevertheless, after reviewing published reports, Andersen et al. (2020) concluded that recording cerebellar activity was possible. Purkinje cells are amongst the largest in the nervous system and cerebellar cortical surface recordings show large positive potentials associated with CFR field potentials of up to 500 μV (Eccles et al., 1967). Recent work using both MEG and EEG techniques support this view. Lin et al. (2019) using optically pumped magnetometers (OPMs), recorded evoked responses from sensors placed over the cerebellum with components at approximately 50 ms and 100 ms in response to an air puff US. Using source analysis techniques they showed that these cerebellar evoked fields were generated from within the posterior cerebellum which they suggested were likely to be of climbing fiber origin.

In our own work we have reported cerebellar evoked potentials (CEPs), produced by vestibular and axial stimuli (Todd et al., 2017; Govender et al., 2020; Todd et al., 2021b) from scalp electrodes placed over the posterior fossa. In contrast we were unable to obtain responses following superficial radial nerve stimuli (Govender et al., 2020), consistent with a limited electrophysiological “view” of the cerebellum. Somatosensory cerebellar evoked responses are likely to be generated in the anterior lobe, and thus some distance from the skull surface. The vestibular and axial CEPs showed large and reproducible differences in amplitudes between subjects and had properties consistent with them being CFRs (Todd et al., 2018; Govender et al., 2020) – the large amplitudes (in some), surface positivity, post discharge inhibition, that is, an inactivation response, and the electrode location over the cerebellum. The supposed cerebellar origin has also been supported by source analysis (Todd et al., 2021b). In addition, it appears possible to record the tonic electrocerebellogram (ECeG: Todd et al., 2018), which has a higher frequency content than cerebral EEG, confirming early reports of high frequency, intrinsic cerebellar oscillations (Adrian, 1935). It is likely that the higher frequencies (> 80 Hz) are more specific for cerebellar activity (Todd et al., 2018) and reduce contamination from other sources such as EMG.

As noted above, CFRs and associated post‐CFR pause (the inactivation response) are of particular interest from a learning theory perspective as it has been suggested that they are the neural signaling pathway for a UR (Albus, 1971). In Todd et al. (2021a) we reported an eye blink conditioning experiment using a mastoid tap US, the latter known to activate vestibular receptors as well as producing an eye blink UR and a robust CEP with an inactivation response, and so a presumed CFR. In the reported experiment the head‐tap US was paired with auditory and visual CSs and did produce eye blink conditioning, albeit weak, which subsequently showed extinction. However, of particular interest, were the observations that showed correlated EMG with post‐CFR pausing in response to the US in the high frequency ECeG, and evidence of a conditioned pause in the ECeG associated with the CR, consistent with the Albus hypothesis.

In the present study we wished to explore recording from the cerebellum and the cerebrum in parallel during classical conditioning using an established method causing robust conditioning. A corneal air puff is a commonly employed US (Lin et al., 2019), but determining latencies following such a stimulus is difficult due to the inherent delays associated with the air puff method. An alternative to the corneal air puff is electrical stimulation of the trigeminal nerve, which has also been employed in studies of the role of the cerebellum in eye blink classical conditioning (e.g., Teo et al., 2009). We wished to determine whether there were characteristic electrophysiological changes arising from the human cerebellum, and what relationship such changes might bear to the evoked reflexes, both UR and CR, as well as parallel changes recorded centrally. To this end, we examined event‐related responses and high frequency power in EEG, ECeG and EMG/EOG activity. Given previous evidence individual differences in eye blink conditioning, we also wished to determine if any such observed electrophysiological changes related to individual performance.

## 2 METHODS

### 2.1 Ethics statement

Fourteen healthy adults (six female, eight male; three left handed, 11 right handed; age range: 30–66) were recruited from the general community and staff and students at the Prince of Wales Hospital, the University of New South Wales and Western Sydney University. Written informed consent was obtained from all participants prior to testing. The study was approved by the local ethics committee (South Eastern Sydney Local Health District Human Research Ethics Committee) and the experiment was performed in accordance with the Declaration of Helenski. No participant had a history of vestibular, hearing or neurological impairment.

### 2.2 Auditory (CS) and electrical (US) stimulation

The CS consisted of 500 ms, 2 kHz sinusoidal auditory tones given at an intensity of ‐ 60 dB re 5 V (~ 80 dB pSPL) bilaterally. Auditory tones were generated using Signal software (version 6.02) and a CED Power1401 interface (Cambridge Electronic Design), and delivered via a custom amplifier and insert earphones. The US consisted of bilateral electrical stimulation of the maxillary branches of the trigeminal nerve. Electrical stimulation was applied beneath the eyes using adhesive electrodes (H69P cloth electrodes, Covidien) and generated using two DS2A isolated stimulators (Digitimer Ltd). Short current pulses (0.1 ms) were used and the sensory thresholds measured independently for the left and right sides. Stimulation levels were initially set to three times the threshold, but then increased if a significant blink was not induced, up to a maximum of 28 mA. Low intensity maxillary nerve stimulation was chosen for subject comfort as well as to reduce stimulus artifact in the electrophysiological recordings.

### 2.3 Electrophysiological recordings

**FIGURE 1:**
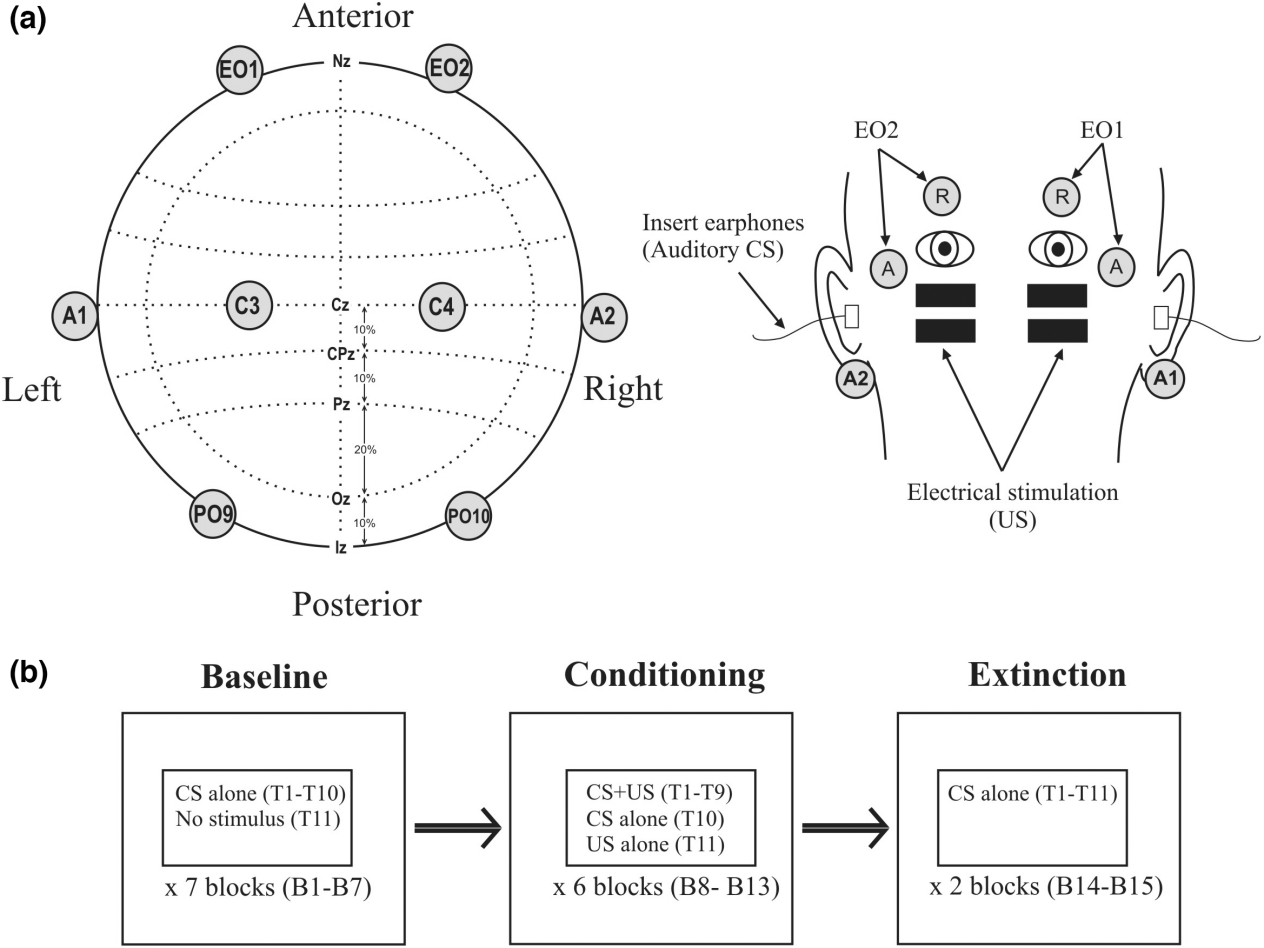
(a) Extraocular (EO1/2), central (C3/4) and cerebellar (PO9/O10) electrode locations used during recordings. Central and cerebellar electrodes were referenced to the ear lobes (A1/2). Both the auditory conditioned stimulus (CS) and unconditioned electrical stimulus (US) were delivered bilaterally. The extraocular electrode montage consisted of active and reference electrode locations (A & R) around the eyes. (b) The consecutive recording sets (baseline, conditioning and extinction), which consisted of fifteen blocks in total (B1–B15) with each block made up of eleven individual trials.

Subjects were seated upright during the recordings and simultaneous measurements were made from extraocular and cephalic locations (Figure 1a). Extraocular activity was recorded bilaterally using a bipolar montage (EMG/EOG: EO1 & EO2) and consisted of active electrodes positioned near the lateral canthus and reference electrodes directly above the eye. Cephalic potentials were recorded from the central areas (EEG: C3 & C4) and over the posterior fossa (ECeG: PO9 and PO10) and referenced to linked ear lobes (A1 & A2). An earthing electrode was placed over the sternoclavicular joint. Recordings were made using a 10–10 customized EEG cap (EASYCAP GmbH) or Ag/AgCl electrodes. For clarity, unrectified averages were bandpass filtered between 8 and 100 Hz to remove EOG and very high frequency spontaneous activity. Recordings over the posterior fossa were localized based on previous mapping and source analysis studies (Govender et al., 2020; Todd et al., 2021b). The total length of the recording epoch was 2.1 s with a 200 ms interval preceding the onset of the auditory conditional stimulus with the electrical unconditioned stimulus given 500 ms later. Signals were amplified (EMG/EOG: × 10,000; EEG and ECeG: × 20,000), filtered (0.5 Hz – 1 kHz) and sampled (10 kHz) using a CED Power1401 and recorded using Signal 6 software (Cambridge Electronic Devices).

### 2.4 Experimental procedure

We used a procedure based upon an adaptation of that of Teo et al. (2009). The recording paradigm for each subject consisted of baseline, conditioning and extinction recording sets (Figure 1b). Each recording set consisted of up to 7 recording blocks, with each block always containing eleven trials (T1–T11). Before conditioning, a baseline set was recorded and consisted of seven blocks (B1–B7) of CS alone trials (T1–T10), with the eleventh trial containing no stimulus (T11). For the conditioning set, six blocks (B8–B13) were recorded with auditory CS and electrical US stimuli combined for the first nine trials (T1–T9: CS + US) and then delivered separately for the tenth (T10: CS alone) and eleventh trials (T11: US alone). After conditioning, an extinction set was carried out consisting of two blocks of CS alone trials (B14–B15). An intertrial interval of 8–12 s was used across recording sets.

### 2.5 Behavioral analysis

We quantified alpha, conditioned, and unconditional response types in each trial based on previously reported criteria (Teo et al., 2009; Todd et al., 2021a). An alpha response (αR) was defined as an EMG/EOG response in EO1/2, which occurred within 200 ms after the onset of the CS. A conditioned response (CR) as an EMG/EOG response in EO1/2, which occurred within 300 ms prior to the onset of the US for CS + US trials and within 300 ms prior and 200 ms after the expected onset of the US for CS alone trials. Finally, an unconditioned response (US) was defined as an EMG/EOG response in EO1/2, which occurred shortly after the US.

### 2.6 Spectral power

Stimulus artifact was removed by linear interpolation and back fill of high frequency power above 80 Hz. Spectral power analyses were performed for the six channels (EO1/2, C3/C4, CB2/4) using the continuous wavelet transform (CWT) as implemented in the MATLAB toolbox (R2019b, Mathworks). In the present analysis a Morlet wavelet was employed at a density of 24 voices per octave over 9 octaves. The CWTs were further transformed to scalograms (time‐frequency images) from the absolute value of the CWT and rescaled to be in dB per voice re 1 μV^2^. Scalograms were computed for all trials, then further split into frequency bands; delta (δ: 1.8 Hz – 4 Hz), theta (θ: 4–7.8 Hz), alpha (α: 7.8–12.5 Hz), beta (β: 13–30 Hz), gamma (γ: 30–80 Hz), ultra‐gamma (u‐γ: 80–160 Hz), very high frequency (VHF: 160–320 Hz) and ultra‐high frequency (UHF: 320–640 kHz). RMS (root mean square) averages were also made using similar bandpass‐filtered frequencies. Bandpass filtering was performed to minimize the effects of EOG.

### 2.7 Data and statistical analysis

Signals were measured from the unrectified, filtered unrectified and spectral power averages. Trials with CRs were realigned manually to the onset of the EOG including 0.4 s prior and 0.5 s following it, using a custom program written in MATLAB 2007b (Mathworks) and reaveraged. In subjects who showed behavioral conditioning, the CR onset latency was measured using unrectified averages. For the central EEG electrodes, the UR was measured after further bandpass filtering (8–100 Hz) as unrectified averages were likely to be contaminated with backspread of EOG. In CS containing trials, long latency N1 and P2 auditory evoked potentials (AEPs) were measured.

Repeated‐measures ANOVAs were carried out on blink CRs for the learning block with BLOCK and TRIAL (US only trials excluded) as within‐subjects factors. For AEPs ANOVAs were employed to compare potentials before and during conditioning, with factors of GROUP (strong vs. weak conditioners – see below) and TRIAL TYPE (CS trials before conditioning versus CS trials during conditioning, with US only trials excluded). ANOVA were also carried out on ECeG power separately for each of the top three bands after segmentation of the epoch before and during conditioning. The epoch segments were as follows: 1–100 ms prior to CS onset; 2–0 to 100 ms of CS, 3–100 to 200 ms of CS, 4–200 to 250 ms of CS, 5–250 to 300 ms of CS, 6–300 to 350 ms of CS, 7–350 to 400 ms of CS, 8–400 to 495 ms of CS, 9–15 to 30 ms after US, 10–50 to 65 ms after US, 11–65 to 140 ms after US, 12–140 to 200 ms after US, 13–200 to 300 ms after US and 14–300 to 400 ms after US. These were designed to sample the whole epoch, including the baseline, but also to increase the resolution at the critical times of expected conditioned and unconditioned responses, and to avoid time of the residual stimulus artifact. There were thus four within‐subjects factors of BLOCK (1–6), TRIAL (1–10, after removal of US only trial types) SIDE and SEGMENT (1–14). Subsequent ANOVAs assessing differences in conditioning also included GROUP (strong vs. weak conditioning) as the between subjects factor. Mean (SD) is reported in the text. In cases using ANOVAs, *p*‐values have been corrected for possible sphericity violations.

### 2.8 Personality assessments

Given the known systematic differences between individuals in ease of conditioning (Eysenck and Levey, 1972), we also administered the “Big five inventory” (BFI‐44), a 44 item questionnaire to measure personality traits of openness, conscientiousness, extraversion, agreeableness and neuroticism (John et al., 2008). Univariate ANOVAs were employed to test for GROUP effects.

## 3 RESULTS

### 3.1 Electrical thresholds and stimulation levels

The mean threshold levels were 5.1 (2.1) mA and 4.8 (1.3) mA, respectively, for the right and left sides. The mean stimulation levels were 16.8 (6.8) mA and 15.5 (6.1) mA, respectively, corresponding to 3.3 times threshold bilaterally. None of the subjects reported any aversive effects of the stimulation.

### 3.2 Behavioral results

**FIGURE 2:**
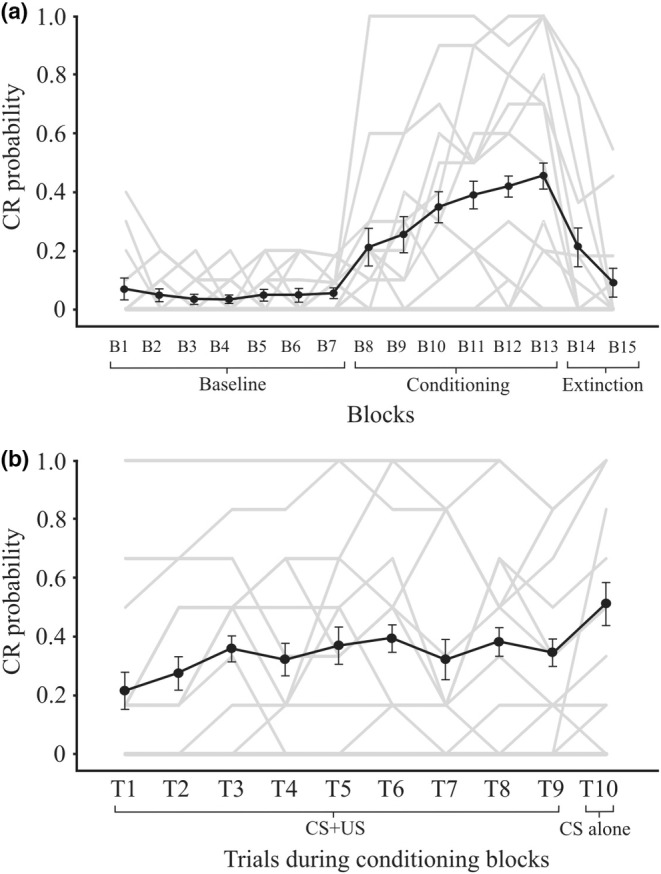
(a) Main effect of block in which CR probability remained low during baseline (B1–B7), increased during conditioning (B8–B13) and decreased with extinction (B14–15). (b) Main effect of trial, which shows CR probability increasing steadily from T1 to T10 during the conditioning blocks. Gray traces reflect individual subject data.

The probability of observing a CR in all trials containing a CS (CS only during baseline control blocks, CS only and CS + US trials in the conditioning blocks, and CS only during the extinction blocks) is given in Figure 2a. This shows that during the baseline observations, there was a small level of spontaneous blinks, about 5%, rising to an overall level of 36% in the conditioning blocks before falling steadily in the two extinction blocks to about 19% (Figure 2a). Within the conditioning blocks, CRs occurred in the first conditioning block at about 21%, rising to about 46% in the last conditioning block, thus exhibiting a clear effect of BLOCK (*F*(5,60) = 8.9, *p* < 0.001) and a weaker but significant effect of TRIAL (*F*(9,108) = 3.6, *p* < 0.05) (Figure 2b).

### 3.3 Electrophysiological results

#### 3.3.1 Grand unrectified means of extraocular, central and cerebellar responses

**FIGURE 3:**
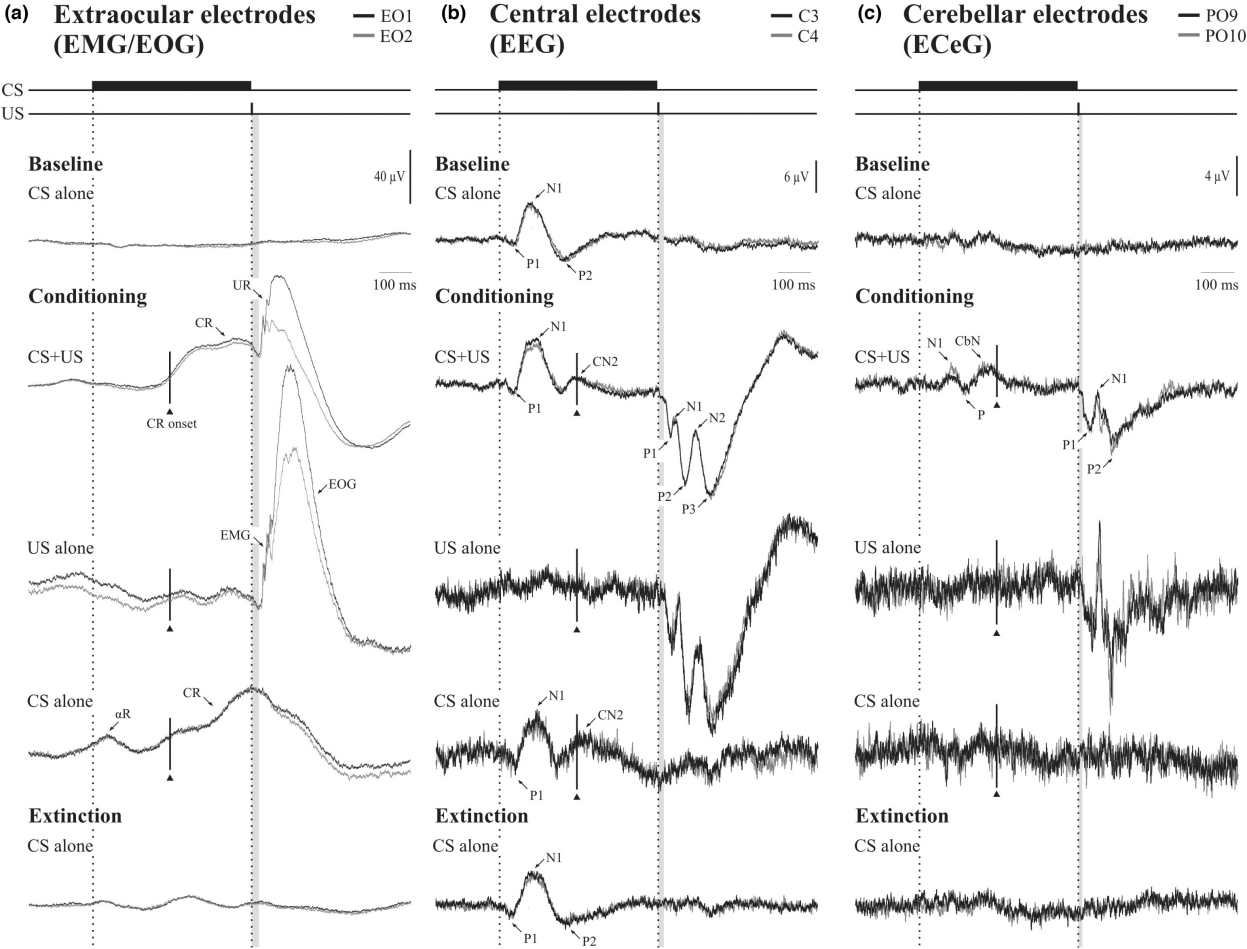
Grand mean unrectified traces from the extraocular (a: EO1 and EO2), central (b: C3’ and C4’) and cerebellar (c: PO9 and PO10) recording locations (*n* = 14). Traces reflect the average across blocks for the baseline (B1–B7), conditioning (B8–B13) and extinction (B14–B15) sets. For the extraocular recordings, a conditioned response (CR) can be observed in the CS + US and CS alone trials during conditioning, whereas the CR was abolished during extinction. An unconditioned response (UR) is also observed in the CS + US and US alone trials and shown to be composed of both EMG and EOG components. A smaller alpha response (αR) was present in CS alone trials during conditioning. For the central recordings, an auditory evoked P1‐N1‐P2 triphasic response was present across all CS containing trials. During conditioning a later conditioned negativity (CN2) became present, which was subsequently abolished during extinction. US containing trials produced short latency evoked potentials that consisted of a series of positive (P1, P2, P3) and negative (N1, N2) peaks. For the cerebellar recordings, a conditioned negativity (CN) was also observed in the CS + US trials. US containing trials produced a triphasic P1‐N1‐P2 response, similar to those observed in the central electrodes. Gray vertical shading indicates the region following US onset in which stimulus artifact was removed ‐ 24 ms (extraocular), 19 ms (central) and 14 ms (cerebellar) and applied to all figures hereafter. The line and solid triangle show the mean onset of the CRs.

Grand means of extraocular, central and cerebellar responses are illustrated in Figure 3. For the ocular responses (Figure 3a), CRs were apparent in the CS + US and CS alone trials, with a mean onset latency of 243 (37) ms in the CS + US trials. The CS + US and US alone trials also exhibited well‐defined URs, which were smaller in the CS + US trials compared to US alone. Both CRs and URs were dominated by the EOG signal, though an EMG burst consistent with the R2 component of the blink reflex was also discernible at the onset of the UR. In the CS alone trial during conditioning, the CR reached a peak close to the timing of the onset of the US. A small αR was also present in the CS alone trials and some evidence of anticipation was apparent in the US alone trials. The conditioned responses were absent in the baseline and extinction sets.

In the case of the central leads (Figure 3b), in the baseline set a well‐defined long latency auditory evoked potential (AEP) was present in which the established P1, N1 and P2 waves were present with latencies of 45.1 (8.2), 97.6 (9.1) and 200.2 (15.6) ms, respectively, and amplitudes 2.1 (1.1), 6.5 (2.4) and 4.3 (2.2) μV, respectively. In the CS + US and CS alone trials during conditioning, however, a second negative deflection appeared shortly after the N1, with a peak latency of 250.7 (14.2) ms in CS + US trials and 267.3 (36.2) in CS alone trials, close to the average CR onset, which disappeared in the extinction blocks. We thus refer to this response as a conditioned negativity, or CN2. When applied to each of the other AEP components across the CS trial type, no effects were obtained for the P1 amplitude or latency but a significant main effect of conditioning was obtained for the N1 amplitude (*F*(1,12) = 12.4, *p* < 0.005; 6.5 (0.52) vs. 8.5 (0.71) μV) indicating that the N1 was enhanced by conditioning. The response to the trigeminal nerve US, the trigeminal evoked potentials (TEPs) consisted of three successive positive–negative deflections P1, N1, P2, N2 and P3 sitting on a slow positive wave (possibly EOG) followed by a slow return to negative. The initial P1 had latencies of 36.0 (6.1) and 39.2 (7.6) ms in the CS + US and US alone trial types, respectively, close to the onset of the UR. As with the ocular responses, the central response to the US was reduced in the CS + US trials compared to the US only.

The cerebellar responses were similar to those recorded centrally in that they consisted of a series of positive and negative deflections to both the CS and US. In the cerebellar case, there was a CEP to the auditory CS consisting of a N1‐P‐CbN complex, with latencies of 108, 146 and 220 ms, respectively, in the grand mean. There was an interaction of WAVE (N1, CbN) by TRIAL TYPE (baseline, conditioning and extinction). Overall, the N1 was unaffected whereas the CbN was enhanced during conditioning only (*F*(2,24) = 3.8, *p* < 0.05). The cerebellar response to the trigeminal nerve US resembled the central US TEP response, consisting of a P1‐N1‐P2 CEP, with latencies of 40, 65 and 106 ms.

#### 3.3.2 High frequency analysis of EMG, EEG versus ECeG in US versus CS + US trials

**FIGURE 4:**
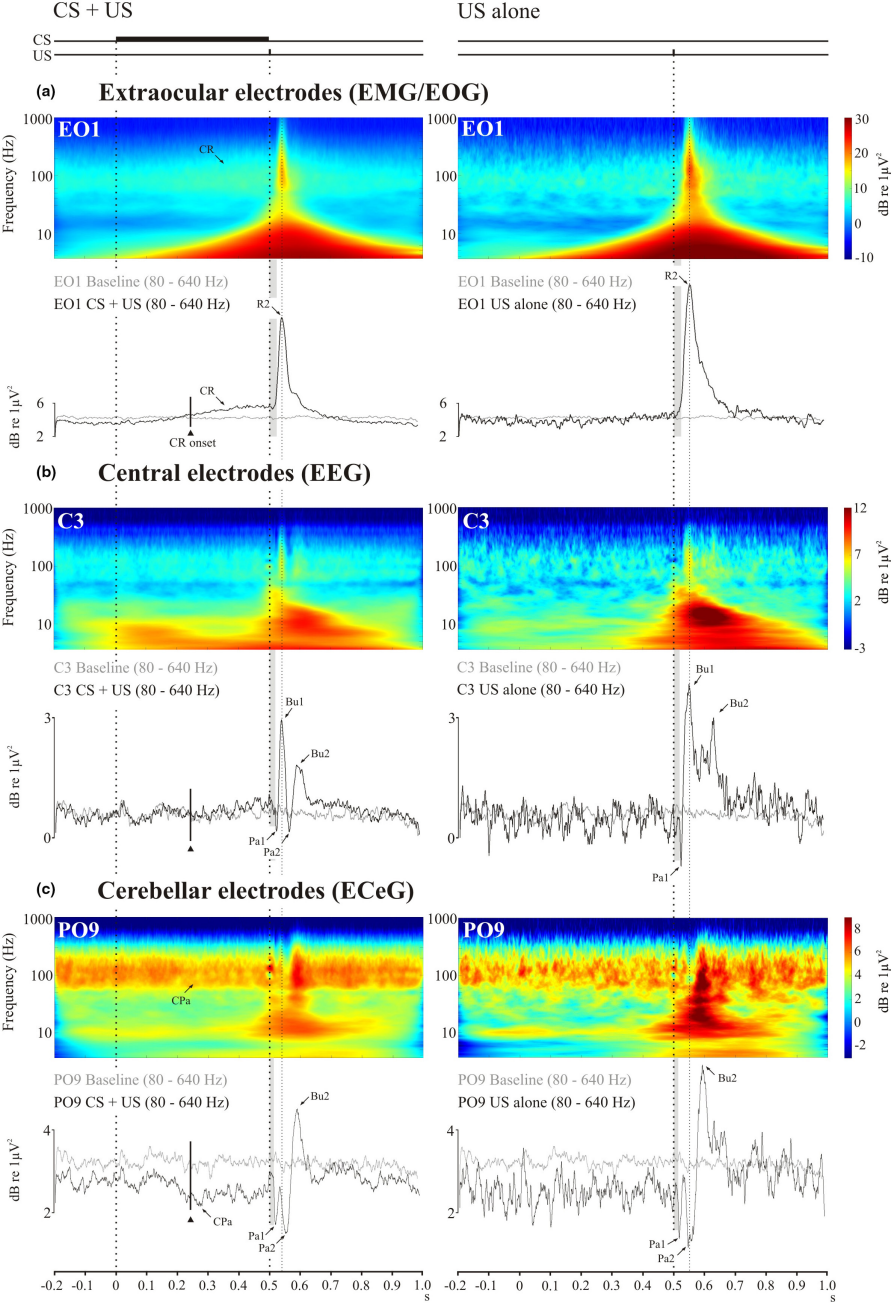
Scalograms and corresponding power traces (80–640 Hz; u‐γ, VHF and UHF) for CS + US (left column) and US alone (right column) trials (*n* = 14). The findings for the baseline condition are shown in light gray. (a) Extraocular, central (b) and cerebellar (c) recordings are shown from the left sided electrodes. Extraocular power demonstrates separation of EMG and EOG components. The conditioned response (CR) is present in CS + US trials and the R2 component of the blink reflex was present in both trial types. Both central and cerebellar electrodes show UR responses consisting of a series of pauses (Pa1 & Pa2) and bursts (Bu1 & Bu2). During CS + US trials a conditioned pause (CPa, 6C) can be observed in PO9 around the onset of the CR (solid triangle).

Figure 4 illustrates scalograms and high frequency power from the top three bands (u‐γ, VHF and UHF) covering 80–640 Hz for the CS + US condition compared against the high frequency power in US condition. This analysis has the advantage of separating the EMG from the associated EOG and reducing any backspread of EOG to the EEG and ECeG leads. In the ocular leads a well‐defined EMG burst was present in response to the US, while the EMG component of the CR was present in the CS + US trials. The underlying R2 origin of the wavelet filtered EMG burst was confirmed with measurements of the onset from high‐pass filtering ocular recordings (see Figure 4). The high frequency EEG and ECeG responses to the US were characterized by a series of pauses and bursts in power, rather than a single burst as in the EMG. For both EEG and ECeG, the pause‐burst series have been labeled Pa1, Bu1, Pa2 and Bu2, which, as illustrated in Figure 4, occur just prior to and following the EMG peak.

**FIGURE 5:**
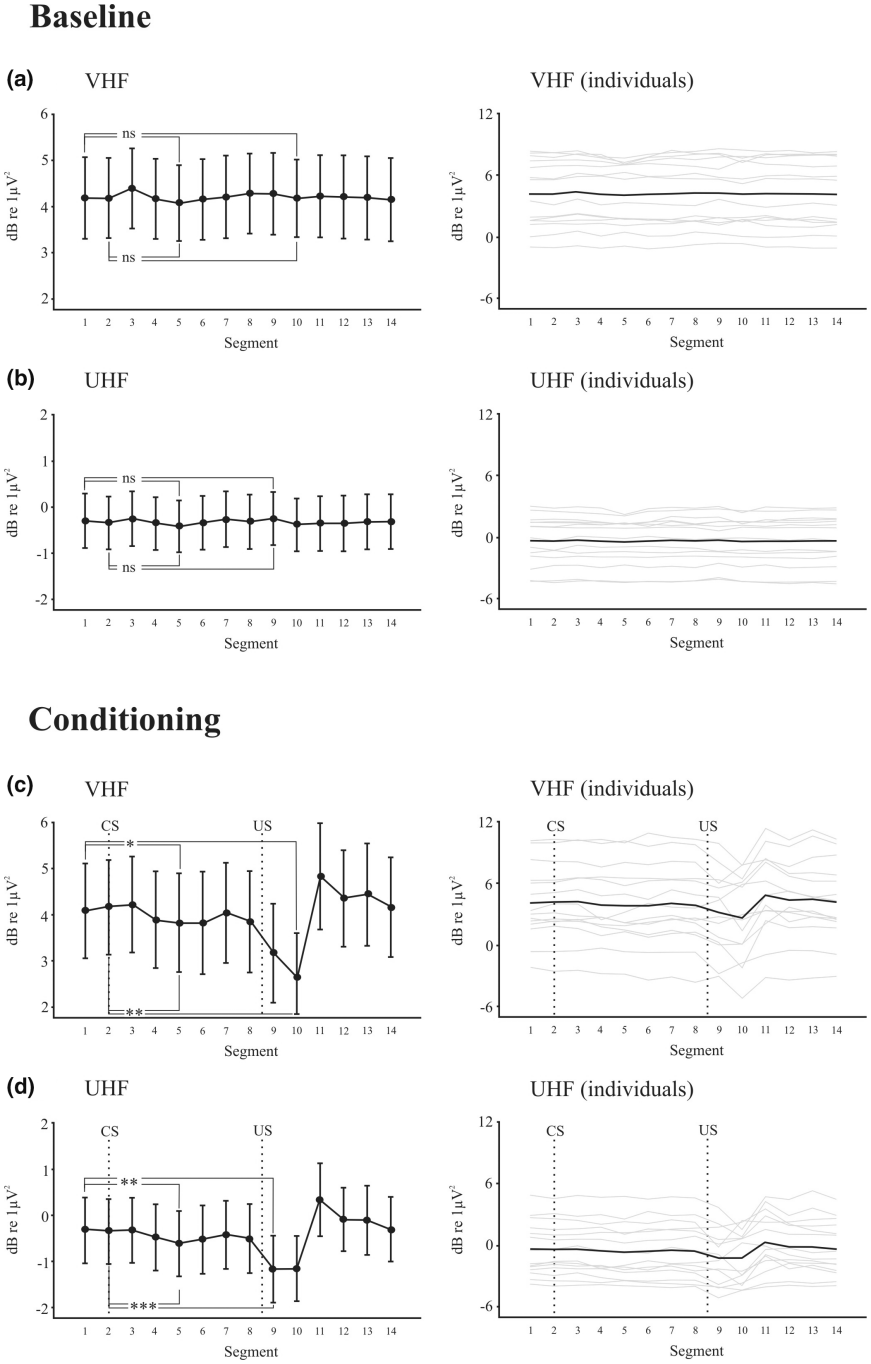
Segment effects for ECeG power (averages across sides) comparing the baseline (CS alone, top two rows) versus learning CS + US blocks bottom two rows for the VHF (a & c) and UHF bands (b & d). Pairwise comparisons of baseline, with conditioned and unconditioned pausing are given * ‐ <0.05, **, <0.01, ***, <0.005, ns = not significant. Individual subject data are shown in the right column (gray traces).

The 14 segments allowed testing for the size of both conditioned and unconditioned changes for comparison (Figure 5). An ECeG power reduction appeared to occur around the time of the CR, indicating a conditioned pause (CPa) in the ECeG (Figure 5c). When repeated‐measures ANOVA was applied for all three bands, main effects were obtained for BLOCK (respectively *F*(5,60) = 6.3, 6.9 and 7.7, with *p* < 0.01, =0.005 and <0.005), for SEGMENT (respectively *F*(13,156) = 5.5, 8.9 and 8.7, with *p* < 0.005, <0.001 and <0.001), and for TRIAL for the u‐γ and VHF bands (respectively *F*(9,108) = 4.8 and 4.7, with *p* < 0.01 and <0.05). An interaction of TRIAL by SEGMENT was also obtained for the VHF and UHF bands (respectively *F*(117,1404) = 2.3 and 2.2, with *p* < 0.05 and <0.05). Both BLOCK and TRIAL effects correspond to a successive drop in power. When the same test was run over the baseline blocks, a main effect of BLOCK was present, which may indicate that BLOCK effect in the learning blocks was not solely due to conditioning. However, neither TRIAL nor SEGMENT effects (Figure 5a, b) were present. The maximum dip in ECeG power during the post US segments for the CS + US trials occurred in segments 9 and 10, corresponding to the power pausing minima Pa1 and Pa2. The maximum dip during the CS segments occurred in segments 5 and 6, that is, between 250 and 300 ms and thus close to the mean onset of the CR. Pairwise comparison of the first two segments, CR‐related segments 5 and 6 and UR‐related segments 9 and 10 indicated significant effects for the VHF and UHF bands (Figure 5c, d), although not the u‐γ band (not illustrated). When the same pairwise comparisons were made for the baseline CS alone blocks, no effects were observed.

#### 3.3.3 Temporal relations of the high frequency EMG, EEG and ECeG components and evoked potentials

**FIGURE 6:**
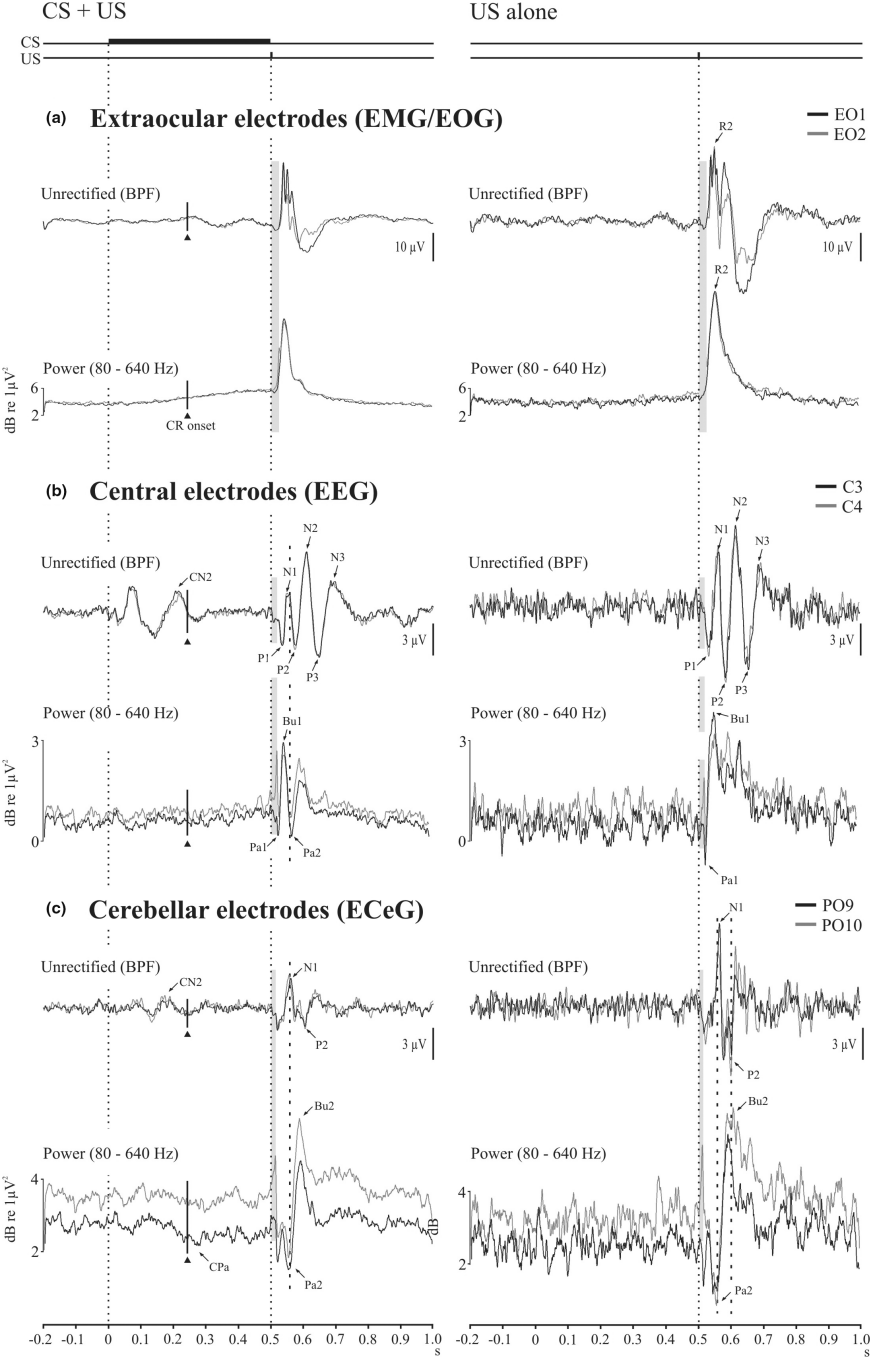
Grand means (*n* = 14) comparing unrectified traces with bandpass filtering (BPF; 8–100 Hz) and corresponding high frequency power traces (80–640 Hz; u‐γ, VHF and UHF) for the extraocular (a), central (b) and cerebellar (c) electrodes. Both filtered and high‐ frequency power traces suppressed EOG contamination of the recorded signals.

Figure 6 shows the waveforms after bandpass filtering (8–640 Hz) as well as power (80–640 Hz) to remove the effects of the EOG. On the left side the CS + US response is shown and on the right, the US. In the EO recordings (Figure 6a), the peak of unrectified mean representation of the EMG is closely aligned with the filtered EMG burst. The mean unrectified EMG onset occurred at 30.2 (6.4) ms consistent with a likely R2 origin. For the central recordings (Figure 6b), the filtering revealed TEP components (i.e., the P1, N1, P2, N2, P3 and N3 waves). For the CS + US trials the TEP N1 aligns approximately with the high frequency EEG P2. The temporal relation between the TEP components and the high frequency EEG pause‐bursts is less well‐defined in the US alone case because the high frequency EEG response is much more diffuse. For the cerebellar recordings (Figure 6c), the temporal relationship between the trigeminal cerebellar evoked potentials (TCEPs) on the unrectified average and ECeG pause‐busting for power was similar to the central case. The unrectified TCEP P1, N1, P2 waves were aligned with the cerebellar high frequency pause‐bursts Pa1, Pa2 and Bu2 with similar latencies of around 20, 58 and 95 ms.

### 3.4 Group effects

#### 3.4.1 Group differences in behavior

There were clear individual differences in conditioning performance. Of the 14 subjects in the sample, half showed individual block effects, determined by their reaching at least 50% CRs in the last learning block. This criterion was used to define a GROUP (*F*(1,12) = 41.3, *p* < 0.001) factor for later analysis. There were no significant effects for threshold or stimulation levels for GROUP. A univariate ANOVA on each of the BFI five measures versus conditionability group, yielded a significant GROUP effect for extraversion (*F*(1,11) = 8.9, *p* < 0.05) with marginal means of 17.4 (1.2) versus 12.0 (1.3), respectively, for the strong versus weak conditioning groups. None of the other measures yielded a significant effect, although there was a trend for the weakly conditioning group to score higher on the neuroticism scale (*F*(1,11) = 2.7, *p* = 0.13) with marginal means of 15.4 (2.2) versus 20.8 (2.4), respectively. There were no effects of age nor sex.

**FIGURE 7:**
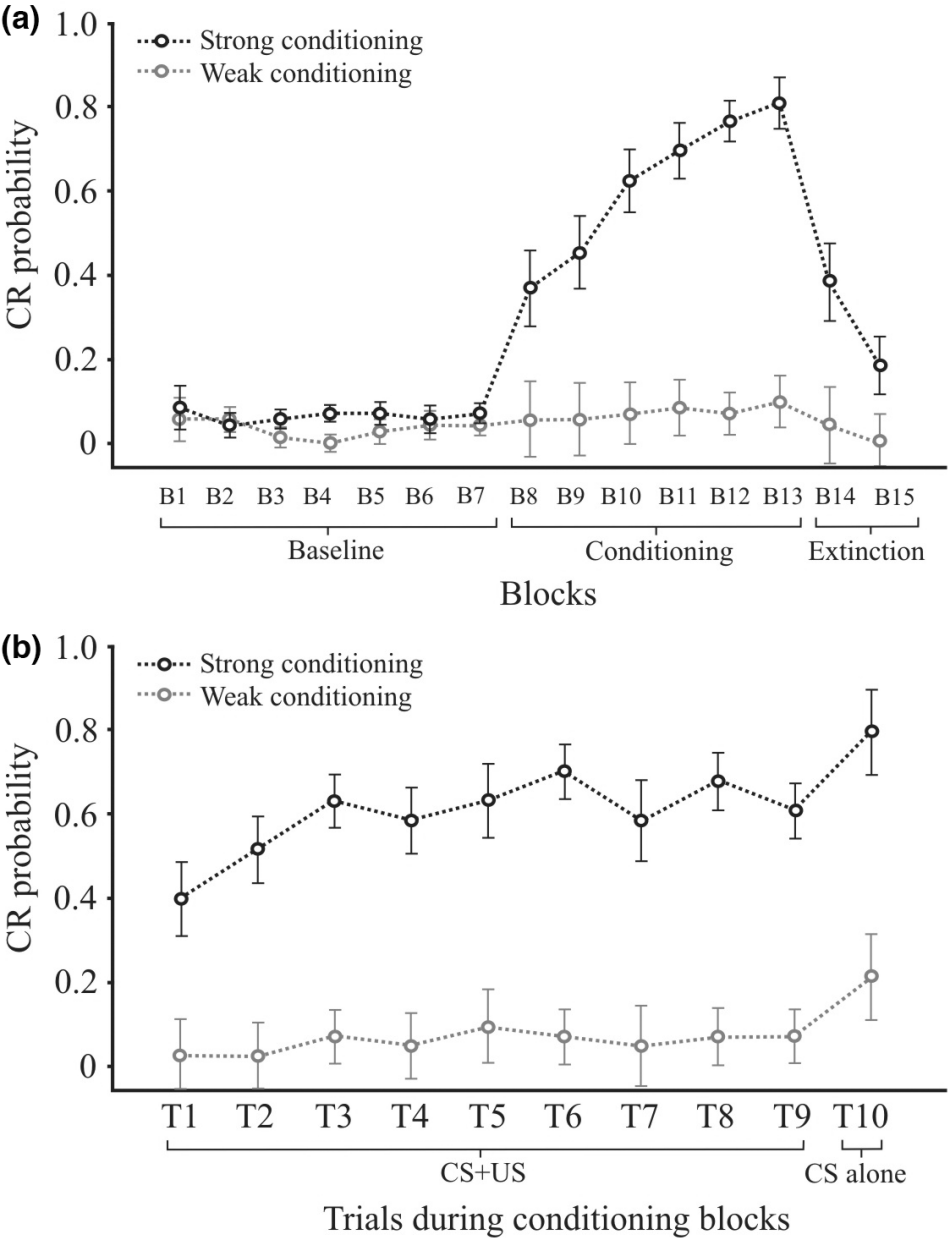
(a) Marked differences in CR probability between the strong (black) and weak conditioning groups. For the strong conditioning group, CR probability increased significantly during conditioning (B8‐B13). (b) Despite no TRIAL by GROUP interaction, CR probability generally increased over trials, more so for the strong conditioning group.

When split by the GROUP factor (Figure 7), the strong conditioning group reached over 80% in the last learning block compared to just over 10% for the weak learner group. The ANOVA carried out on the learning blocks gave a main effect of GROUP (*F*(1,12) = 41.3, *p* <. 001) and an interaction of BLOCK by GROUP (*F*(5,60) = 6.4, *p* = 0.001), but no TRIAL by GROUP interaction.

#### 3.4.2 Group differences in the grand means

**FIGURE 8:**
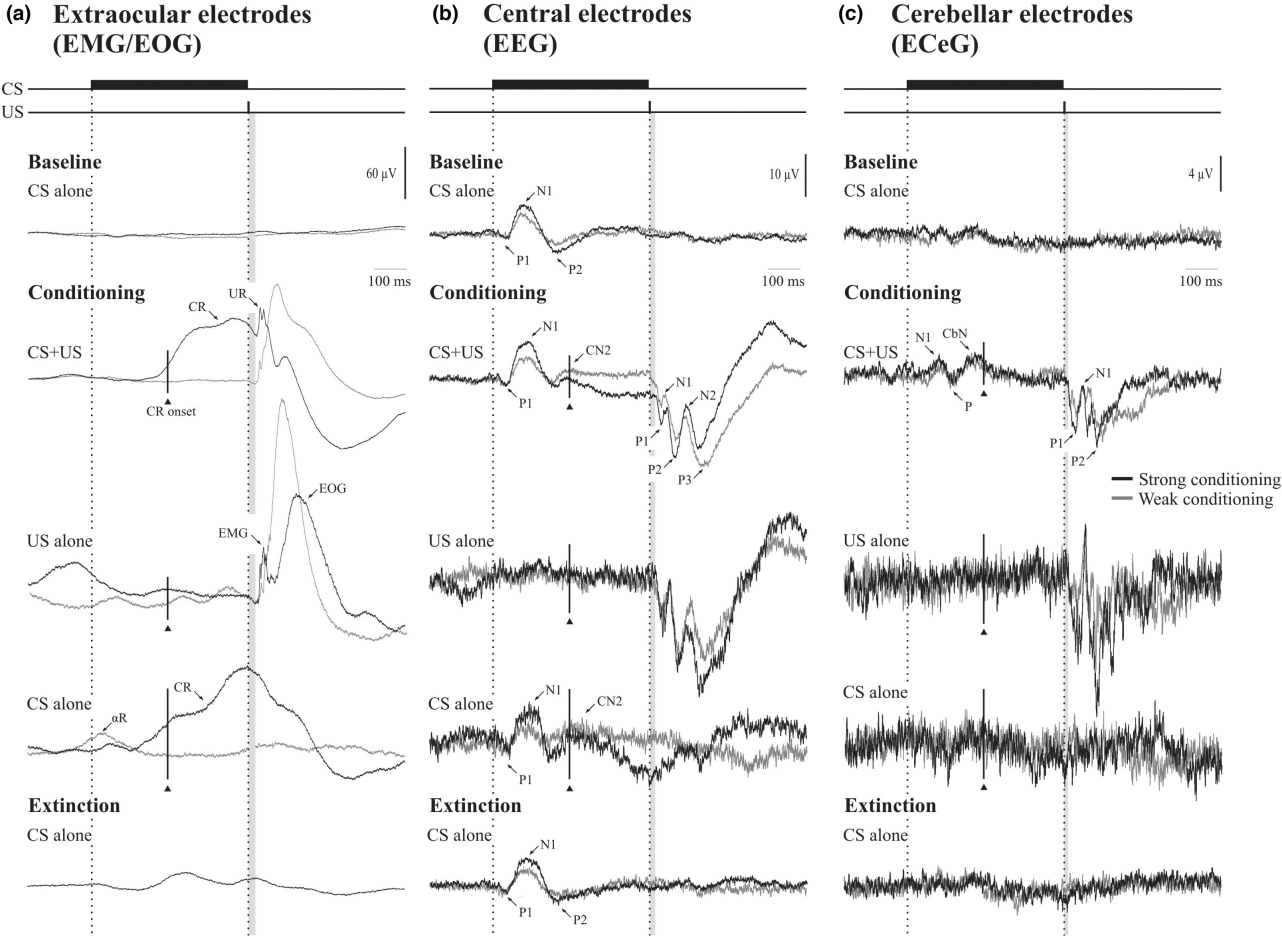
Grand mean unrectified traces from the extraocular (a), central (b) and cerebellar (c) recording locations split by the strong (*n* = 7; black traces) and weak (*n* = 7; gray traces) conditioning groups. There was a clear difference between the groups in the conditioned ocular responses (part A, CS + US), as expected by definition of the two groups. Differences for the EEG and ECeG electrodes were less marked. Note the appearance of the CN2 peak in the EEG (central electrodes) and also the cerebellar electrodes for both groups. The pre‐US positivity for the central electrodes for the strongly conditioning group, might represent distant EOG. Traces reflect the average across blocks for the baseline (CS alone, B1‐B7), conditioning (B8‐B13) and extinction (B14‐B15) sets.

The grand means for the ocular, central and cerebellar recording locations for the two groups are shown in Figure 8. By definition, the groups are separated by the frequency of CRs in the CS only and CS + US trials. The weakly conditioning group do, however, exhibit a small CR at the time that the US would have occurred in the CS only trials, as well as αRs (Figure 8a). They also showed a larger and earlier response to the US alone. Comparing the responses in the central electrodes, differences emerge for the two groups (Figure 8b, middle section). In the control condition, prior to conditioning, the N1‐P2 AEPs were larger in the strongly conditioning group. During learning the N1 showed greater enhancement in the conditioning group, manifest in a GROUP main effect (*F*(1,12) = 9.0, *p* < 0.05, 5.9 (0.79) vs. 9.2 (0.79) μV) over both baseline and CS + US trials. The CN2 was not significantly different between the two groups (*F*(1,12) = 0.8, *p* > 0.05, 4.7 (0.86) vs. 3.6 (0.86) μV). For the cerebellar recordings, there was no difference in N1 and CbN potentials between the groups (*F*(1,12) = 1.1, *p* > 0.05).

#### 3.4.3 Group differences in power after realignment with EOG

**FIGURE 9:**
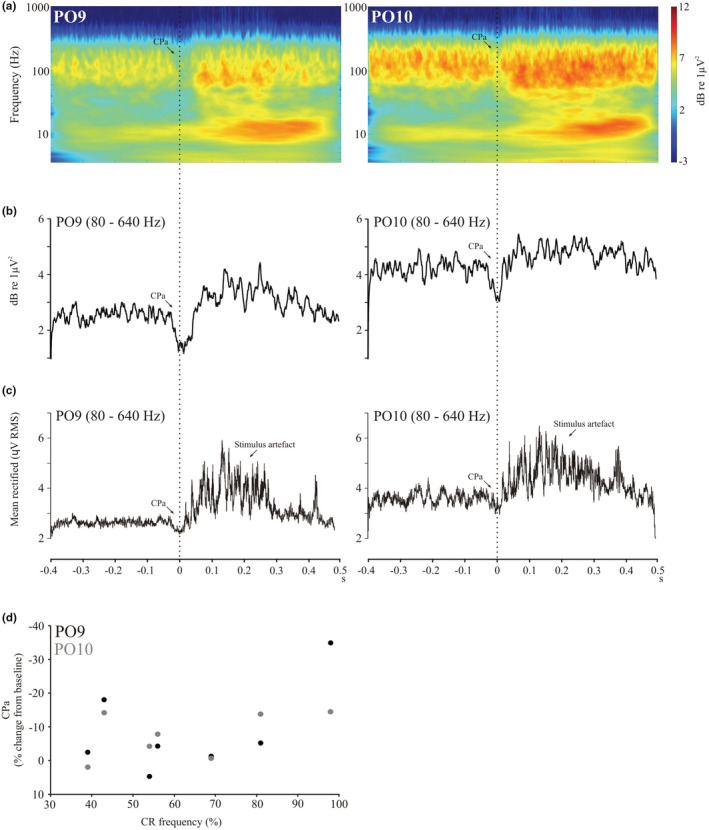
Scalograms (a), high frequency power (b) and high frequency RMS traces (c) for the strong conditioning group realigned to the onset of the EOG. Realigned traces shows conditioning pausing (CPa) in both PO9 (left column) and PO10 (right column) cerebellar electrodes. The correlation between the magnitude of CPa and the frequency of CR trials in the strong conditioning group for the two sides did not reach significance (d).

We also investigated any possible effects for those trials in which a CR was observed, after realigning the traces to the start of the EOG response. Scalograms (Figure 9a), HF power and RMS traces (Figure 9b,c) for the strongly conditioning group after realignment showed a pause in power with a maximum dip close to the onset of EOG, which appeared larger for PO9 than for PO10. When run as a repeated‐measures ANOVA, the effect of SIDE did not reach significance for either HF power or RMS analyses (respectively *F*(1,6) = 5.0, *p* = 0.067 and *F*(1,6) = 5.0, *p* = 0.66). We also tested for a relationship between the magnitude of conditioned pause and CR frequency (Figure 9d), which despite showing a trend, did not reach significance.

#### 3.4.4 Group differences in the high frequency power

**FIGURE 10:**
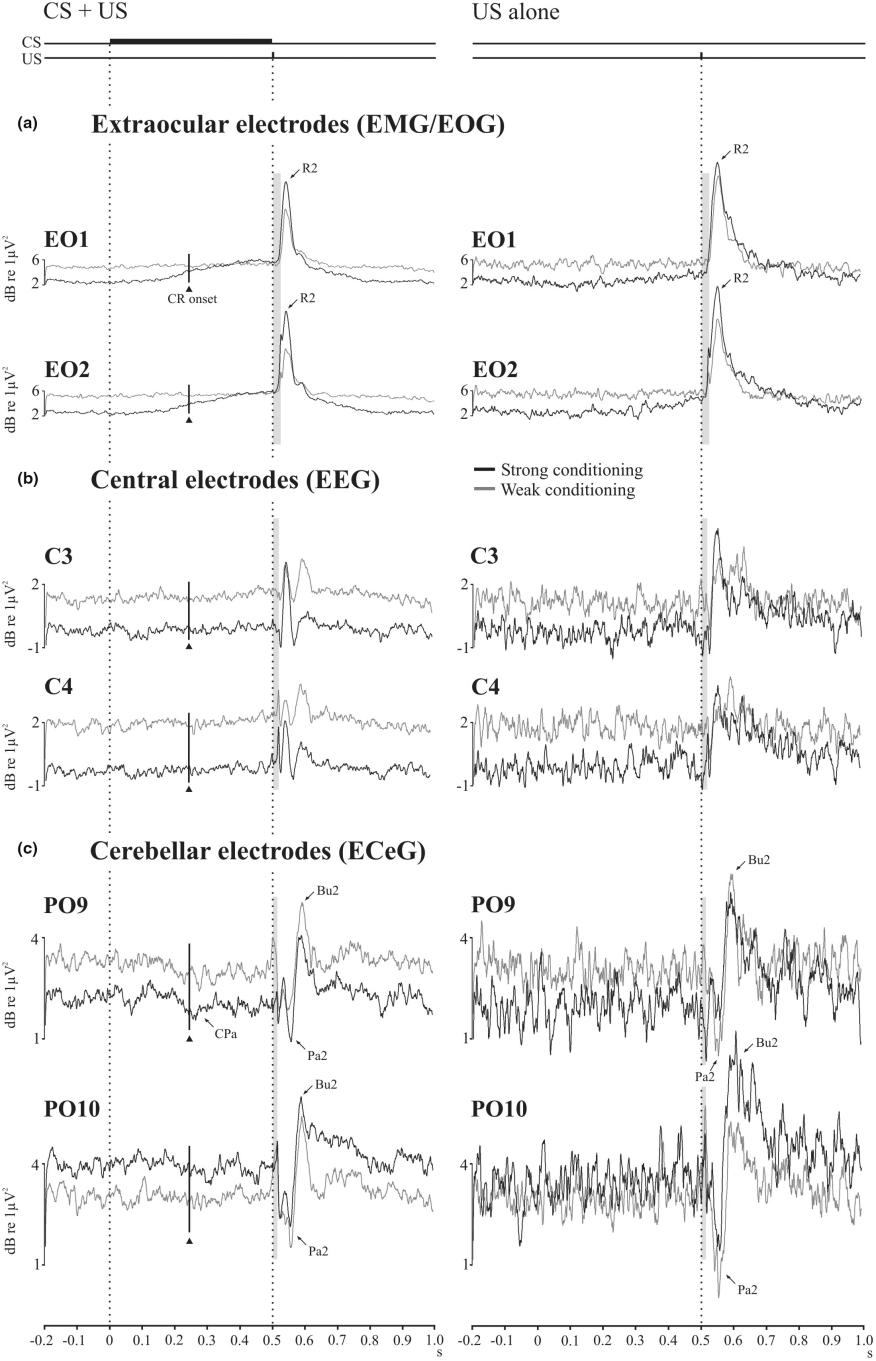
High frequency power (80–640 Hz; u‐gamma, VHF and UHF) from the extraocular (a), central (b) and cerebellar (c) recording locations for the strong (*n* = 7; black traces) and weak (*n* = 7; gray traces) conditioning groups. The left column shows the US+CS case, the right the US alone. The strongly conditioning group showed EMG prior to the onset of the UR peak (EO1, EO2, left side). The two groups, however, did not differ significantly for the ECeG averages. The traces reflect the average across blocks for the conditioning set (B8–B13).

Figure 10 illustrates the extracted high frequency power from the top three bands for extraocular, central and cerebellar electrodes split by GROUP. The strong conditioning group show conditioned EMG beginning prior to the onset of the R2 (Figure 10a). The strong conditioning group also exhibit some left–right asymmetries in the unconditioned EMG, EEG and ECeG burst pausing. However, when applied to the segmented high frequency ECeG power, there were no significant GROUP effects, nor GROUP interactions with SEGMENT in any of the high frequency bands so that, irrespective of behavioral conditioning, the high frequency conditioning changes were not significantly different for the two groups in the ECeG.

## 4 DISCUSSION

Considering the sample as a whole, we obtained three main results. The first was the presence of the hypothesized pausing of the high frequency ECeG, a partial confirmation of the prior Albus hypothesis in intact humans. The second was the observation of a conditioned negativity (CN2) in the central leads aligned to the onset of the CR. The third was the confirmation that personality may have profound effects on conditioning under our conditions. However, there was no difference between the strong and weak conditioning group for either the central or cerebellar activity. We conclude that conditioned cerebellar pausing may be necessary, but is not sufficient, to produce overt behavioral conditioning, implying the existence of another central mechanism. Our results are thus broadly consistent with recent extensions of the Marr‐Albus‐Ito conceptualization of cerebellar learning (Sanger et al., 2020; Kawato et al., 2021) and provide new insights into its manifestation in humans.

Following the application of the trigeminal nerve US we observed pause‐bursting, in fact a double pause‐burst, the Pa1‐Bu1‐Pa2‐Bu2, in the high frequency ECeG power, consistent with a post‐CF “inactivation response.” The cerebellar double pause‐burst UR was closely aligned with the P1‐N1‐P2 trigeminal CEP (TCEP), which, in turn, was aligned with the central P1‐N1‐P2 TEP components and its high frequency EEG power manifestation, also in the form of a double pause‐burst. These cooccurred with the R2 related EMG burst component of the UR, thus each representing independent but correlated evoked and induced ocular, cerebral and cerebellar manifestations of the UR. The latencies of the N1‐P2 components of P1‐N1‐P2 trigeminal CEP at about 65 and 105 ms, were similar to the latencies of the early and late MEG components reported by Lin et al. (2019) from eye‐puff stimulation, consistent with a trigeminal origin. The significance of the double pause‐burst in the ECeG may be related to its alignment with the rising and falling edges of the EMG. Whereas the first pause‐burst precedes the R2 related EMG peak, the second follows and may be associated with the blink being generated by two different muscle groups affecting both the eyelid and extraocular muscles (Schlag et al., 1983). Our peri‐ocular montage, arranged to minimize stimulus artifact, was not optimized to record from the extraocular eye muscles.

In addition to the UR‐related pausing, we observed conditioned pausing in the high frequency (VHF/UHF) ECeG power preceding the US after conditioning, consistent with a conditioned pause in PC simple spike activity as hypothesized by Albus. This was manifest as a phasic CR‐related drop in power compared to the baseline (pre CS onset) activity. The magnitude of this conditioned power reduction at about 0.5–1 dB is not large in absolute terms and is about 1/3 of the magnitude of the subsequent unconditioned pause components. Our present data also showed significant tonic reductions in ECeG power, manifest as significant BLOCK and TRIAL effects with a tonic decrease of about 2 dB compared to the control blocks. The BLOCK effect may in part be due factors other than conditioning, such as change in alertness over the course of a set of experimental blocks as cerebellar spontaneous activity is known to be modulated by states of arousal (Canto et al., 2017). The TRIAL effect though appears to be a genuine conditioning effect as it was not present in the control blocks. A conditioned tonic reduction in the high frequency ECeG may correspond to a general facilitation given the inhibitory action of Purkinje cells (Eccles et al., 1967).

The second notable result was the appearance of a conditioned negativity (CN2) in cerebral recordings. The cerebral CN2 peaked at 250 ms, close to the average onset of the CR at 244 ms, while the cerebellar CbN peaked at about 220 ms, preceding the cerebral CN2 by about 30 ms. The CbN was, in turn, preceded by an earlier negativity, giving the appearance of a double negative. One possibility for the origin of this conditioned cerebellar double negative wave is that it may be a manifestation of a conditioned climbing fiber response prior to the conditioned pause (ten Brinke et al. 2019) and thus a conditioned analogue of the unconditioned climbing fiber response and post‐CF pause. It has been well‐established in animal models that there are conditioned changes in complex spike activity during conditioning (Rasmussen et al. 2014) and that conditioned climbing fiber responses tend to occur as doubles within the first 200 ms from the CS onset (ten Brinke et al. 2019). It has been argued that these conditioned CFRs, in addition to playing a role in the initiation of the conditioned pause, may also act as an onset marker signal for sensory‐evoked motor initiation (Tsutsumi et al. 2020). This includes other cerebral motor centres involved in movement initiation, timing and selection, such as frontal cortex and basal ganglia, which are targets of cerebellar nuclear output (Bostan and Strick 2018). The cerebral CN2 on the other hand, may be a manifestation of an early component contingent negative variation (CNV). This is associated with motor timing and known to have frontal cortical and basal ganglia generators (Cui et al., 2000; Macar et al., 1999; Casini and Vidal, 2011) and also to be associated with cerebellar activation (Nagai et al., 2004). In the first reports it was accepted that for both conditioned reflexive and voluntary action the early components of the CNV arose out of the second vertex negativity following the conditional or warning stimulus, prior to the later slower negativity developing before the unconditional or imperative stimulus (Walter et al., 1964).

Although the experiment produced successful conditioning, it was highly variable across individuals to the extent two distinct groups were clearly defined by the probability of CRs in Block 6. There were no differences in thresholds or stimulation levels between the groups, so this cannot be the explanation. There were also other commonalities. Both groups showed the EMG UR and associated cerebral and cerebellar URs, including the high frequency double pause‐bursting, which is likely associated with a CFR. In the case of the responses to the CS, both groups also showed evidence of electrophysiological conditioning in the form of the cerebellar conditioned pause and the prior cerebellar and cerebral conditioned negativities associated with the movement onset, and likely early CNV component manifest as the CN2. Despite these similarities, only one group showed overt conditioning. There were some differences in the two groups' responses to the auditory CS, including in the control condition N1 prior to conditioning. Notably, however, and the third principal result, was the difference between the two groups in their scores using the extraversion‐introversion scale, a result, which had been established by Eysenck and others in the early days of human eye blink conditioning (Eysenck, 1965). It should be noted that there is prior evidence of a personality difference in AEPs (Stelmack and Wilson, 1982) and also that the differences we recorded were not due to differing sensitivity to the stimuli used (Rammsayer, 2004). Eysenck pointed out that variability in conditioning performance could be reduced by the employment of very strong USs (Eysenck and Levey, 1972). Our US intensity was in part chosen to reduce the electrical stimulus artifact, but with the outcome that it revealed the effect of personality in inhibition of the conditioned response. Given the importance of stimulus intensity, it is surprising how often this is not quantitated, at least in terms of a ratio to the threshold level, which can be applied to most stimuli, including the air puff. Our stimulus was thus on average 10.4 dB above threshold. Teo et al. (2009) used 5 times threshold (14 dB) and Hoffland et al. (2012) 7–10 times threshold (17–20 dB). Eysenck and Levey (1972) reported that a 6 dB increase in the US intensity was sufficient to abolish personality effects.

Thus, taken together, the above results suggest that while cerebellar conditioning may be necessary, it appears to not be sufficient to produce overt behavioral conditioning in humans, and an additional, noncerebellar, learning mechanism must be involved in human conditioning. As noted in the Introduction, the original Marr‐Albus theory had not considered a role for cerebrum, accepted as a weakness in the modern reviews 50 years on, and there is evidence for a role of both the hippocampus and basal ganglia (Bostan and Strick, 2018). Our finding of a difference on extraversion‐introversion also points to the basal ganglia as a likely candidate for the additional central mechanism of “inhibition” because of the established correlation between extraversion and dopamine (DA) function, particularly for the projection to mesolimbic areas but also mesostriatal (Depue and Collins, 1999; Rammsayer, 2004; Smillie et al., 2010). Dopamine has been proposed to be a modulator of incentive motivation (Depue and Collins, 1999) and also plays a central role on basal ganglia function. Due to the presence of two major DA receptor types, dopamine has a differential effect on the direct and indirect pathways via the D1 and D2 receptors, respectively (Gerfen and Surmeier, 2011). In models of basal ganglia learning of action selection the two pathways are considered to be involved in action selection and control, respectively (Gillies and Arbuthnott, 2000), so that it might be supposed that extroverts versus introverts may have differential activity of the direct versus indirect pathways, and hence different levels action selection versus control. However, despite spontaneous blinking being reduced in Parkinson's disease, Parkinsonian patients do not show impairment of eye blink conditioning (Daum et al., 1996; Sommer et al., 1999).

The cerebellum and basal ganglia are now established as being anatomically directly connected via two‐way disynaptic projections: from the cerebellum via the deep nuclei and thalamus to the striatum (primarily D2 zones) and from the basal ganglia via the subthalamic nucleus and pontine nuclei to the mossy fiber input pathway (Bostan and Strick, 2018). The striatal input from the thalamus to the D2 striatal zone could provide the signaling pathway by which conditioned climbing fiber responses signal onsets for sensory‐evoked motor initiation (Tsutsumi et al., 2020), but then the basal ganglia controls whether an overt action results (Gillies and Arbuthnott, 2000). There is a long standing debate about the differential roles of the cerebellum and basal ganglia in motor timing (Breska and Ivry, 2018). The present data are consistent with the view that that the primary motor timer is the cerebellum, while the principal role of the basal ganglia is in movement selection/control. During classical conditioning, the cerebellum may thus serve as one component of a broader network that subserves movement timing and learning. Our findings fit with Ito's model of cerebellar function, with LTD (long term depression) following Purkinje neuron activation (Ito, 2000, 2001; Sanger et al., 2020; Kawato et al., 2021). In Ito's model, repeated pairings of granule cell activity and Purkinje cell activity, depresses the input efficiency of the granule cell axons. This is consistent with our observations of a reduction in ECeG and demonstrates the potential value of noninvasive cerebellar electrophysiology as a tool for investigating its mechanisms in humans.

## FUNDING INFORMATION

Research was supported by the Australian Research Council (ARC) Discovery Grant DP210100552.

## CONFLICT OF INTEREST STATEMENT

The authors report no conflict of interest, financial, or otherwise.
